# Case Report and Literature Review—From Ultrasound to Genotype: Periventricular Nodular Heterotopia

**DOI:** 10.1155/crog/6563701

**Published:** 2025-12-22

**Authors:** B. Novillo-Del Álamo, R. Gómez-Portero, A. Martínez-Varea, R. Quiroga, J. Rubio-Moll, R. Llorens-Salvador, A. Zuñiga-Cabrera, B. Marcos-Puig

**Affiliations:** ^1^ Department of Obstetrics and Gynecology, La Fe University and Polytechnic Hospital, Valencia, Spain, hospital-lafe.com; ^2^ Department of Medicine, CEU Cardenal Herrera University, Castellón de la Plana, Spain, uchceu.es; ^3^ Division of Paediatric Radiology, La Fe University and Polytechnic Hospital, Valencia, Spain, hospital-lafe.com; ^4^ Department of Genetics, La Fe University and Polytechnic Hospital, Valencia, Spain, hospital-lafe.com; ^5^ Department of Pediatrics, Obstetrics and Gynecology, Faculty of Medicine, University of Valencia, Valencia, Spain, uv.es

**Keywords:** heterotopia, magnetic resonance, prenatal, ultrasound

## Abstract

**Background and Aims:**

Periventricular nodular heterotopia is an unusual disorder caused by a neuronal migration disorder.

**Methods:**

A case report and narrative review of the literature were carried out.

**Results:**

This pathology involves multiple systemic manifestations, mainly neurological (seizures) and cardiovascular (valve insufficiency). Only 186 periventricular nodular heterotopia patients have been described in the literature. The present case report is one of the scarce cases diagnosed prenatally by ultrasound. Postnatal genetic test revealed the newborn was heterozygous for the FLNA gene variant mutation, associated with an X‐linked dominant inheritance pattern with ventricular heterotopia.

**Conclusion:**

This study underlines that comprehensive prenatal diagnosis helps with paternal counseling, newborn management, and preconception counseling.

## 1. Introduction

Periventricular nodular heterotopia (PVNH) Type 1 is a disease caused by a mutation of the Filamin A (FLNA) gene (MIM # 300049), located on chromosome Xq28 [1, 2]. Filaminopathies are generally X‐linked dominant pathologies [2, 3]. Therefore, adult patients are usually females, as males normally have prenatal or early postnatal death [2, 4].

PVNH is a neuronal migration disorder. Neurons fail to migrate from the marginal periventricular area to their final location in the cortex, creating nodules of ectopic neurons [5]. It is a heterogeneous and rare disorder characterized by mainly neurological (seizures and intellectual disability of variable severity) and cardiovascular (patent ductus arteriosus, severe aortic valve insufficiency, dilation of aorta, and pulmonary hypertension) manifestations [2, 6, 7], but also pulmonary (respiratory distress, hyperinflation, and atelectasis) [8], intestinal (pseudo‐obstructions and intestinal malrotation), craniofacial (hypertelorism, low‐set ears, and micrognathia), ophthalmological (strabismus and orbital fullness), and other systemic symptoms (joint hypermobility, deep vein thrombosis, thrombocytopenia, etc.) [2].

A case report of a patient referred to the tertiary La Fe University and Polytechnic Hospital, Valencia, Spain, is detailed. This hospital has a multidisciplinary committee for prenatal and postnatal counseling and follow‐up that includes, among others, subspecialized obstetricians in ultrasound and maternal–fetal medicine, pediatric radiologists, geneticists, and neonatologists.

## 2. Case Report

A 25‐year‐old patient, a native of Morocco, was referred from a regional hospital to La Fe University and Polytechnic Hospital, Valencia, Spain, for pregnancy follow‐up since the 20th week of gestation. The patient had a history of two prior spontaneous miscarriages in the first trimester. She had no other relevant personal or familial medical history. Her partner was a 43‐year‐old man, a native of Morocco, with no relevant personal or familial medical history. The couple suffered a year of primary infertility of unknown etiology before this pregnancy, which was achieved by in vitro fertilization using their own gametes without preimplantation genetic diagnosis.

**Figure 1:**
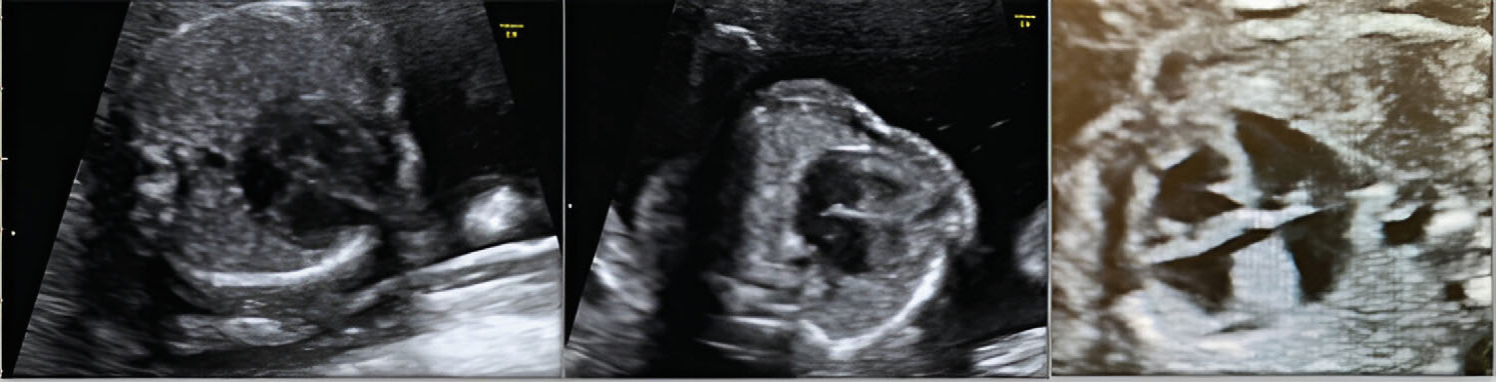
Echocardiogram performed during Week 22 of pregnancy.

The 12‐week ultrasound was within normal limits. The patient displayed a low risk for aneuploidies in the first‐trimester combined screening. However, neurological and cardiac abnormalities were detected at a morphological 20‐week ultrasound, so they were referred to La Fe University and Polytechnic Hospital, Valencia, Spain. An advanced fetal ultrasound was carried out at Week 22. An echocardiography was performed with the following findings: aortopulmonary disproportion and atrioventricular valve dysplasia (Figure 1). A neurosonography was also executed, and it revealed multiple bilateral periventricular nodular subependymal heterotopias, mega cisterna magna, and bilateral ventriculomegaly.

**Figure 2:**
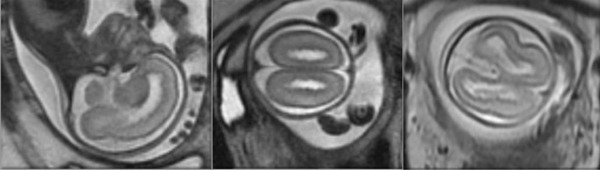
Magnetic resonance performed during Week 23 of pregnancy.

A fetal magnetic resonance imaging (MRI) was carried out during Week 23 to complete the neurosonography information. It revealed multiple subependymal nodular bilateral periventricular heterotopias as well as bilateral ventriculomegaly with frontal predominance (Figure 2).

These prenatal findings suggested that these neurological and cardiological abnormalities could be related to the FLNA mutation. In order to continue with the diagnostic process, an amniocentesis was proposed to the patient and her partner. However, the patient did not want to undergo an invasive test during pregnancy due to personal convictions, and the couple decided to continue with the pregnancy. Therefore, the genetic diagnosis could not be made until the postpartum period.

**Figure 3:**
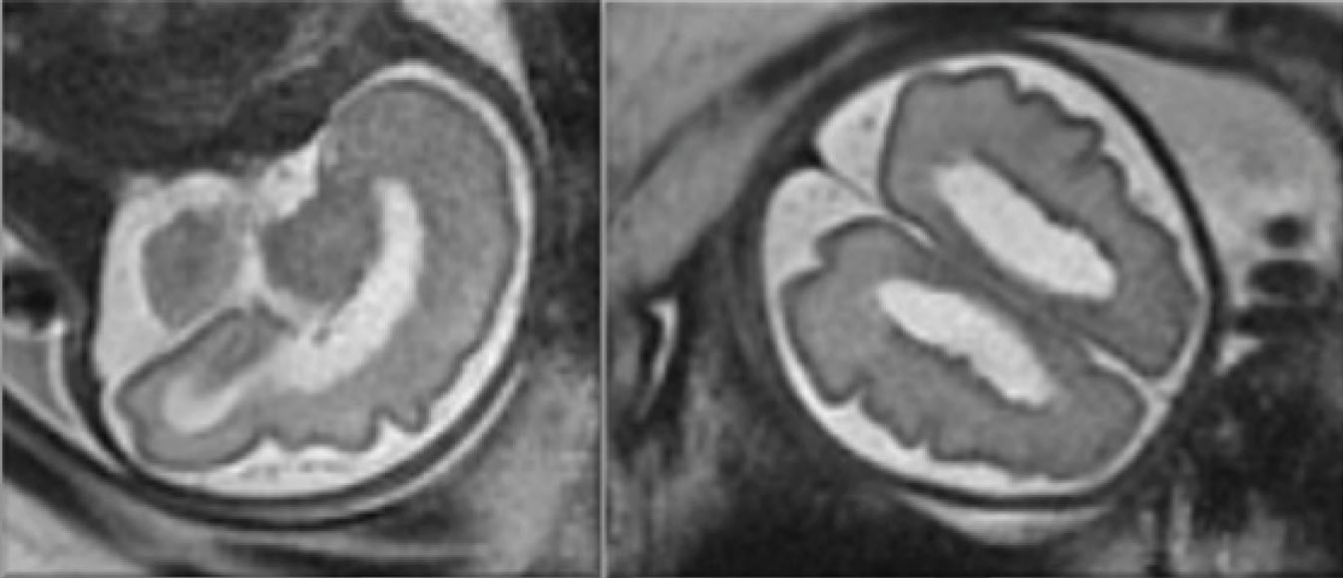
Magnetic resonance performed during Week 29 of pregnancy.

A new MRI was performed in Week 29 in order to seek the evolution of the abnormalities (Figure 3). An ultrasound at 34 weeks of pregnancy revealed normal fetal growth and a normal fetal Doppler study. The fetal abnormalities, including PVNHs, bilateral ventriculomegaly, and atrioventricular valve dysplasia, remained stable compared to previous studies.

The patient underwent a spontaneous onset of labor and had a vaginal delivery at 40th weeks of gestation. The female newborn of 3160 g (percentile 33 for pediatric nomograms) was asymptomatic at birth and was breastfed uneventfully. The anatomical findings diagnosed prenatally were confirmed by physical examination and complementary tests, which included echocardiography, transfontanellar ultrasound, and electroencephalogram.

The echocardiogram showed normal anatomical relations between the cardiac cavities, with normal pulmonary and systemic venous drainage. A wide atrial septal defect of 14 mm was observed. Thickened and redundant atrioventricular valves were visualized, with mitral valve insufficiency. The aortic leaflet presented an asymmetric trivalve with a good opening and no insufficiency. There was evidence of some small ventricular septal defects with little pressure shunt. The left aortic arch had a joint exit of the first two trunks, after which the distal aorta lost some caliber globally without presenting coarctation.

The transfontanellar ultrasound demonstrated multiple bilateral PVNHs of subependymal location. The corpus callosum was present but with decreased biometry (33 mm), and findings related to hypoplasia of the corpus callosum, especially of the splenium. Mega cisterna magna was also visualized.

A strictly normal electroencephalogram was seen. Continuous background wakefulness activity in the form of *activité moyenne*, symmetrical, without epileptiform activity.

At 2 months of age, the baby was admitted to the intensive care unit due to cardiac decompensation due to mild biventricular dysfunction, with favorable evolution using digoxin. She has not presented subsequent seizures. Her neurodevelopment is age‐appropriate, with good motor, cognitive, and communication development.

A genetic study was requested. The newborn was heterozygous for FLNA gene variant mutation c.7898_7900delGGG (p.Gly2633del; NM_001110556.2). It is described as “likely pathogenic” and associated with an X‐linked dominant inheritance pattern with ventricular heterotopia. This mutation has been previously reported in two unrelated female patients with bilateral PVNH [9]. The whole exome was sequenced to detect the mutation, and it was confirmed by Sanger sequencing. This last technique was also performed on the parents, resulting in normal in both. Thus, the mutation apparently arose de novo in their daughter. There is no a priori higher risk of recurrence in the couple′s future offspring. However, there could be, exceptionally, a germline mosaicism in one of the parents, which could increase the risk of recurrence. Therefore, in the gonad of one of the parents, there could be a cell line with the mutation, which is not detectable in blood studies. For this reason, despite the low risk of recurrence, in the event of a future gestation, geneticists recommend prenatal targeted genetic diagnosis.

## 3. Discussion

This case report details a case of a rare filaminopathy. Only 186 PVNH patients have been described in the literature [2]. In a recently published review, the authors studied the various phenotypic characteristics of these affected patients [2]. They concluded that 72.7% of the patients have neurologic manifestations, 48.1% cardiovascular, 26.7% skeletal, 18.2% pulmonary, 16.6% cutaneous, 15% craniofacial, 10.7% gastrointestinal, 10.2% ophthalmological, and 7.5% hematological manifestations [2]. Neuropsychiatric disease has also been described [10]. Seizures are characteristically linked to PVNH [2, 11], with a broad spectrum of epilepsy phenotypes [12].

The present case reports a prenatal diagnosis of PVNH, which is unusual. The patient′s anonymity has been preserved at all times, and she gave her consent to the authors for the publication of her case for academic purposes.

Usually, PVNH is diagnosed postnatally, in an MRI performed on patients looking for a cause of seizures that have started around 10–30 years old [13]. PVNH is estimated to be the cause of seizures in 11%–20% of epileptic patients with cortical malformations [14]. Prenatal diagnosis of PVNH is exceptional, and when it is detected, it is usually through MRI as a casual finding. PVNH is rarely diagnosed prenatally [13], as in the present case report.

In a review of 11 cases, both ultrasound by expert sonographers and an MRI between 28 and 34 weeks of gestation were performed. The patients were referred due to ultrasound abnormalities such as ventricular dilation, increased fluid in the posterior fossa, and corpus callosum agenesis. In 36.4% of cases, ultrasound failed to diagnose periventricular heterotopia prenatally [13]. Therefore, the authors conclude that PVNH is probably underdiagnosed in routine ultrasounds [13]. MRI could be an important and complementary contribution to the diagnosis when any brain alteration has been detected.

Another study with a larger number of cases described an association of PVNH and ventriculomegaly with dysmorphic frontal horns (60%) and anomalies of the posterior fossa (73.3%) [15]. An explanation for the ventriculomegaly due to PVNH could be due to a malfunction in the reabsorption of the cerebrospinal fluid by the ependymal layers of the ventricles covered by the heterotopias [16]. Two variants of PVNH have been described: diffuse and nondiffuse PVNH. FLNA mutations were found in 54.5% of cases with diffuse PVNH. Additional cortical malformations were observed exclusively in nondiffuse PVNH (60%). Thus, diffuse PVNH has a better postnatal prognosis [15]. Finding a cause for ventriculomegaly can be a determining factor in deciding whether or not to continue with the pregnancy. MRI has an essential role in that situation [16]. Therefore, MRI could be useful for completing the brain study and discovering associations between different malformations. However, in the case of the presented patient, MRI did not add more information to that already obtained by neurosonography, but it rejected more associations.

In an article, Sahinoglu et al. conclude that PVNH should be suspected in the presence of irregular ventricular walls on axial view and irregular square‐shaped lateral ventricles on coronal view [17]. These indications could be useful for subspecialized obstetricians in ultrasound, as a way of simplifying the suspicion and, later, completing it with more studies.

The usefulness of performing an MRI on newborns is discussed if we already have a recent prenatal MRI, since no major changes occur in such a short time and the technique is equally valid intrauterus. Literature shows that PVNH demonstrates very similar appearances in prenatal and postnatal MRI [13]. Thus, it might be more efficient to wait for its completion.

There are some cases reported in the literature that describe the correlation between mothers and fetuses with PVNH [13, 18]. However, in these reported cases, there is a wide variety of symptoms, even with the same mutation and image, due to variable gene expression, X‐inactivation, and mosaicism [18]. In a case report, the authors describe a mother who has a fetus with corpus callosum alteration, subependymal heterotopia, and mega cisterna magna. The FLNA mutation was confirmed in a postmortem analysis. This mother had PVNH on MRI despite being asymptomatic. From her second pregnancy, a female neonate was born with PVNH and was asymptomatic until the publication date [18]. In the present case report, the mother was completely asymptomatic, not carrying the mutation in her DNA, so no MRI was performed on her.

The newborn from the present case report was asymptomatic at the time of writing this article, because the development of symptoms usually occurs later. However, thanks to the prenatal study and postnatal genetic confirmation, a close follow‐up will be done—the neonatology service decided to carry out 6‐monthly consultations (increasable if necessary) for early detection of symptoms, as well as genetic advice for future offspring.

## 4. Conclusions

This case report and narrative review of the literature underline the importance of a complete prenatal diagnosis that assists in paternal counseling, newborn management, and preconception counseling. PVNH is a disorder that involves not only systemic manifestations, mainly neurological and cardiovascular, but also other systemic symptoms. Thanks to the prenatal diagnosis and postnatal genetic confirmation, a close follow‐up will be done for early detection of symptoms, as well as genetic advice for future offspring.

**Nomenclature**
PVNH: periventricular nodular heterotopia
FLNA: Filamin A
MRI: magnetic resonance imaging

## Disclosure

All authors have read and agreed to the published version of the manuscript. M‐V.A. and N‐D.Á.B. had full access to all of the data in this study and take complete responsibility for the integrity of the data and the accuracy of the data analysis. They affirm that this manuscript is an honest, accurate, and transparent account of the study being reported, that no important aspects of the study have been omitted, and that any discrepancies from the study as planned have been explained.

## Conflicts of Interest

The authors declare no conflicts of interest.

## Author Contributions

G‐P.R., R‐M.J., L‐S.R., and Z‐C.A. carried out the medical assistance. N‐D.Á.B. wrote the original draft. M‐V.A., Q.R., and M‐P.B. performed valuable improvements in the original draft.

## Funding

No funding was received for this manuscript.

## Data Availability

The authors confirm that all the data supporting the findings of this study are included in the article.
